# Genomic analysis of a novel nontoxigenic *Corynebacterium diphtheriae* strain isolated from a cancer patient

**DOI:** 10.1016/j.nmni.2019.100544

**Published:** 2019-04-09

**Authors:** N.D. Ramdhan, J. Blom, I.C. Sutcliffe, P.M.A. Pereira-Ribeiro, C.S. Santos, A.L. Mattos-Guaraldi, A. Burkovski, V. Sangal

**Affiliations:** 1) Faculty of Health and Life Sciences, Northumbria University, Newcastle upon Tyne, England, UK; 2) Bioinformatics and Systems Biology, Justus-Liebig-Universität, Gießen, Germany; 3) Laboratory of Diphtheria and Corynebacteria of Clinical Relevance-LDCIC, Faculty of Medical Sciences, Rio de Janeiro State University–UERJ, Rio de Janeiro, Brazil; 4) Department of Microbiology, Friedrich-Alexander-Universität Erlangen-Nürnberg, Erlangen, Germany

**Keywords:** Adenoid cystic carcinoma, *Corynebacterium diphtheriae*, invasive infection, nontoxigenic, virulence

## Abstract

The genome of a novel nontoxigenic *Corynebacterium diphtheriae,* strain 5015, isolated from a patient with adenoid cystic carcinoma was sequenced and compared with 117 publically available genomes. This strain is phylogenetically distinct and lacks virulence genes encoding the toxin, BigA and Sdr-like adhesins. Strain 5015 possesses *spaD*-type and *spaH*-type pilus gene clusters with a loss of some gene functions, and 31 unique genes that need molecular characterization to understand their potential role in virulence characteristics.

Nontoxigenic *Corynebacterium diphtheriae* strains have recently emerged as a major cause of invasive infections such as endocarditis, osteomyelitis and septic arthritis among patients [1], [2], [3]. Diphtheria toxoid vaccine induces an antibody response against the toxin [4] and hence is effective against toxigenic *C. diphtheriae* strains. The *tox* gene is present on lysogenizing corynephages which are absent from most nontoxigenic strains [5]. However, some nontoxigenic strains possess inactive *tox* genes with frameshift mutations, known as nontoxigenic *tox* gene–bearing *C. diphtheriae* [6], [7], [8].

A nontoxigenic *C. diphtheriae,* strain 5015, was isolated in Rio de Janeiro, Brazil, from a case of osteomyelitis in the frontal bone of a 41-year-old woman with adenoid cystic carcinoma in the nasal region [9]. In this study, the genome of strain 5015 was sequenced and compared against 117 published *C. diphtheriae* genomes [8].

*C. diphtheriae* strain 5015 was cultured in 5 mL brain–heart infusion broth incubated at 37°C for 16 hours in a shaking incubator. Genomic DNA was extracted from 2 mL of the culture using an UltraClean Microbial DNA Isolation Kit (MoBio, USA) and was sequenced using a 2 × 300 bp Reagent kit v3 on an Illumina MiSeq instrument (Illumina, USA). A total of 16,345,980 paired-end reads were assembled into 51 contigs of >500 bp in size (∼677-fold coverage) using SPAdes 3.9.0 [10]. The size of the assembly is 2.48 Mb (average G+C content 53.7 mol%), which was annotated by the National Center for Biotechnology Information (NCBI) Prokaryotic Genome Annotation Pipeline [11]. The annotated genome sequence of *C. diphtheriae* strain 5015 has been submitted to the DNA Data Bank of Japan, European Molecular Biology Laboratory, and GenBank databases and is publicly available under accession number MSIS00000000.

The draft genome was annotated with 2459 genes, including nine ribosomal RNA (a complete 16S ribosomal RNA, two partial 23S sequences and six partial 5S sequences), 52 transfer RNA, three noncoding RNA and 146 pseudogenes. The repetitive nature of the *rrn* operons is noted to cause gaps in the draft assembly. Of the 2249 coding sequences, 1584 were assigned a known function or a protein family, 615 encoded hypothetical proteins, 46 encoded transposases and four encoded integrases. A phylogenetic tree based on 1261 concatenated core genes using EDGAR [12] clearly separated strain 5015 from other *C. diphtheriae* isolates (Fig. 1). The multilocus sequence typing (MLST) profile of the strain was extracted from the genome assembly using MLST 2.0 [13]. Strain 5015 belongs to a novel sequence type (ST), with the nearest ST in the MLST database being ST149, a double locus variant (Supplementary Table S1). A phylogenetic tree was calculated from the concatenated sequence alignment of 586 reference STs from the MLST database (https://pubmlst.org/cdiphtheriae/) after excluding the sites with missing data following the best-fit GTR+I+G4 substitution model using IQ-Tree [14] and was visualized using iTOL [15]. Consistent with the core genomic tree, strain 5015 is distinct but is more closely related to STs 201, 274, 427 and 579 than ST149 at the nucleotide sequence level (Supplementary Fig. S1).

**Fig. 1:**
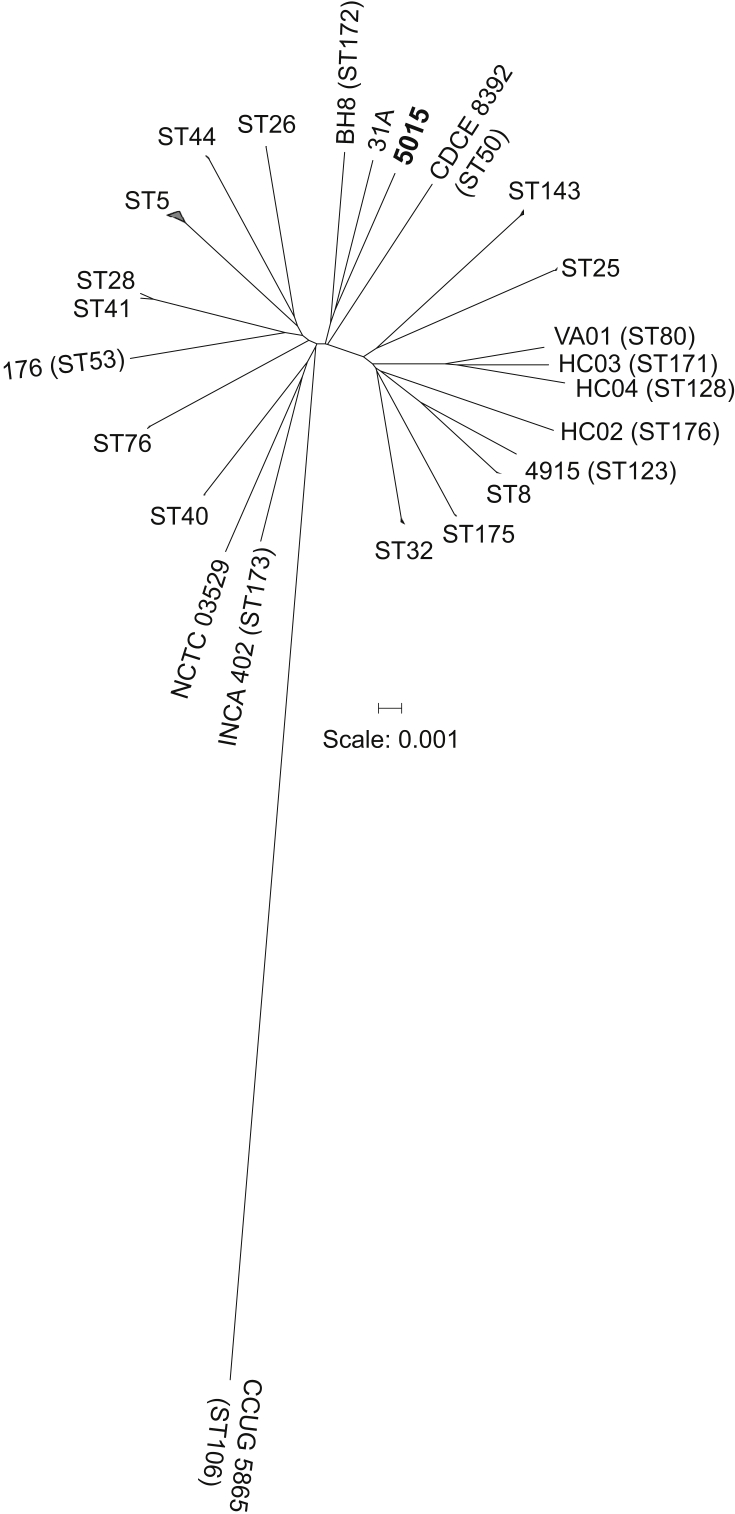
Radial phylogenetic tree from concatenated protein sequences of 118 *Corynebacterium diphtheriae* strains. Nodes containing multiple strains belonging to same STs are collapsed and ST designations are mentioned where known. Scale bar represents number of substitutions per site. ST, sequence type.

Protein BLAST (National Center for Biotechnology Information; https://blast.ncbi.nlm.nih.gov/Blast.cgi) searches confirmed the absence of the *tox* gene (DIP0222), the surface-anchored fimbrial associated protein (SpaA: DIP2066), two adhesins (BigA (DIP2014) and Sdr-family related adhesin (DIP2093)) and SpaA type pili in strain 5015. The *spaD* and *spaH* type pilus gene clusters are present but the *spaD* and *spaG* genes are pseudogenes in these clusters, respectively (Fig. 2). Each of these pili is involved in interactions with laryngeal cells [16], [17]. The *spaD* gene encodes the major pilin subunit in *spaD* pili [17], [18], and it is possible that the interaction with laryngeal cells occurs through homodimeric or heterodimeric SpaE/SpaF proteins, as suggested for the SpaBC cluster in *C. ulcerans* [19], [20]. SpaG is a minor pilin, the base subunit in SpaH pili responsible for anchoring the pilus to the cell wall [17], [21], [22]. The absence of the SpaG subunit may result in the extracellular secretion of SpaH type pili, as predicted for SpaA type pili in some *C. diphtheriae* strains [8], [17]. Strain 5015 exhibited higher adhesive and virulence characteristics than the reference strains American Type Culture Collection (ATCC; Manassas, VA, USA) 27010^T^ (nontoxigenic) and ATCC 27012 (toxigenic), with 100% mortality in a *Caenorhabditis elegans* model [9]. This strain also showed significantly higher intracellular survival in THP-1 and RAW 264.7 macrophages than other strains, and induced arthritis and osteomyelitis in the Swiss Webster mice model [9].

**Fig. 2:**
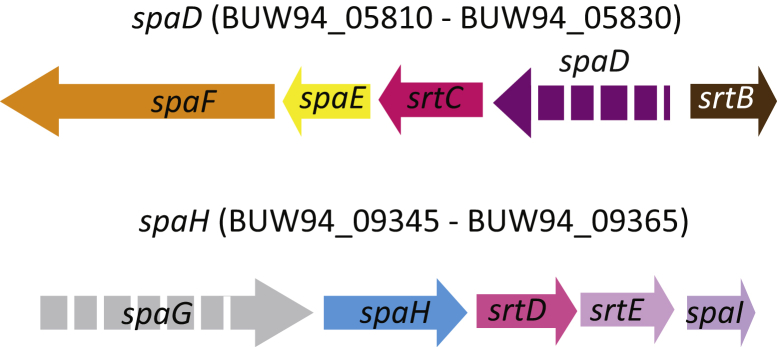
Structure and organization of pilus gene clusters in *Corynebacterium diphtheriae* strain 5015. Pseudogenes are shown by broken arrows; direction of arrow indicates orientation of coding sequence. Not to scale.

Strain ATCC 27012 (C7 β tox^+^) was potentially derived from ATCC 27010^T^ (C7 tox^−^) by treatment with the temperate β corynephage [23], [24]. ATCC 27012 (C7 β tox^+^; GenBank accession no. CP003210.1) also possess *spaD* and *SpaH* type pili, with *spaG* also being a pseudogene in the *spaH* gene cluster [18]. In addition to an intact *spaD* gene cluster, the *bigA* adhesin and *tox* genes are present in ATCC 27012. Toxin production is regulated by iron [25], so we compared the genes involved in iron metabolism in these strains. Both the ATCC 27012 and 5015 strains possess genes encoding iron uptake and transport proteins, including Irp6A-C (DIP0108-DIP0110), DIP0582-0586, HmuT-V (DIP0626-0628) and DIP1059-1062, and lack ChtC-CirA (DIP0522-DIP0523) and ChtAB (DIP1519-DIP1520). Haemoglobin binding protein HtaA (DIP0625) is a pseudogene in ATCC 27012 (C7 β tox^+^), but HtaB (DIP0624) and HtaC (DIP0629) proteins are present. All three of these genes are pseudogenes in strain 5015. A gene-set calculation using EDGAR [12] identified 31 genes unique to strain 5015 among *C. diphtheriae* strains (Supplementary Table S2). However, most of these genes encode hypothetical proteins that are not known to be involved in any virulence-associated activity that would explain the increased virulence in strain 5015. The gene-set calculation between strains 5015 and ATCC 27012 (C7 β tox^+^) revealed 230 additional genes that are present in strain 5015 and absent from ATCC 27012 (Supplementary Table S3). Conversely, 347 genes are present in strain ATCC 27012 and absent from strain 5015 (Supplementary Table S4). Again, a majority of these genes encode hypothetical proteins without any obvious involvement in virulence properties. It is possible that some of these uncharacterized proteins are responsible for the increased virulence of strain 5015 in the *C. elegans* model.

In summary, *C. diphtheriae* strain 5015, isolated from a frontal bone biopsy sample taken from a cancer patient with adenoid cystic carcinoma in the nasal region, is distinct from other *C. diphtheriae* strains and belongs to a novel ST. This strain is nontoxigenic and possesses *spaD* and *SpaH* gene clusters, although it lacks the genes encoding the major pilin subunit and the minor (basal) subunit in these clusters, respectively. Despite the absence of the *tox* gene and key subunits in both the pilus gene clusters and other adhesins, this strain was previously characterized to be more virulent than nontoxigenic ATCC 27010^T^ and toxigenic ATCC 27012. Some of the 31 uncharacterized genes that are unique to this strain may contribute to this enhanced virulence, along with other genes present in strain 5015 and absent from strains ATCC 27010^T^ and ATCC 27012. Therefore, molecular studies are required to characterize the function of these proteins.

## Conflict of Interest

None declared.
