# Supplementary material for: Genomic analysis of a novel nontoxigenic Corynebacterium diphtheriae strain isolated from a cancer patient
Source: New Microbes New Infect. 2019 Apr 9;30:100544. doi: 10.1016/j.nmni.2019.100544 (PMC6488682; doi:10.1016/j.nmni.2019.100544)
Supplement: Multimedia component 1 [file mmc1.pdf]

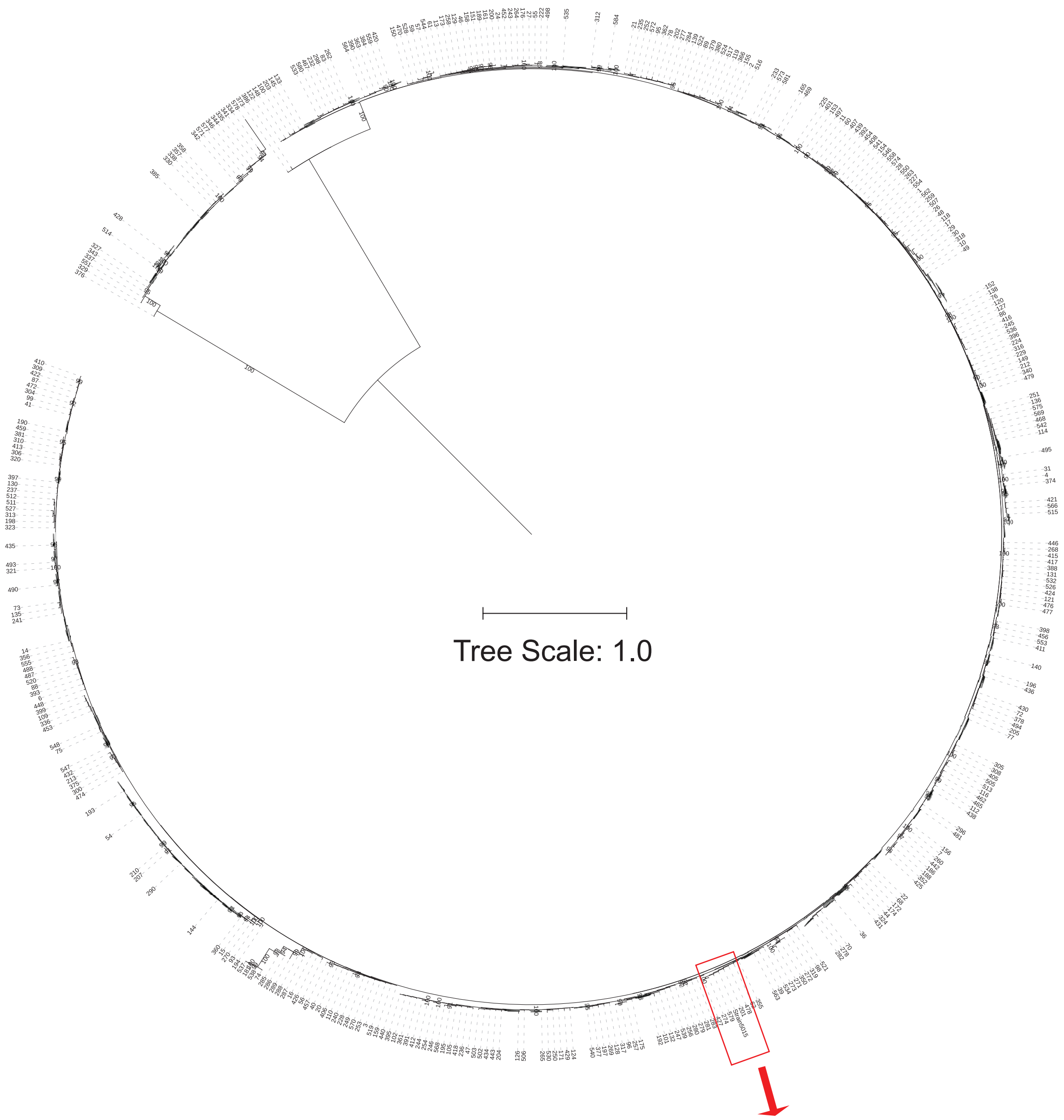

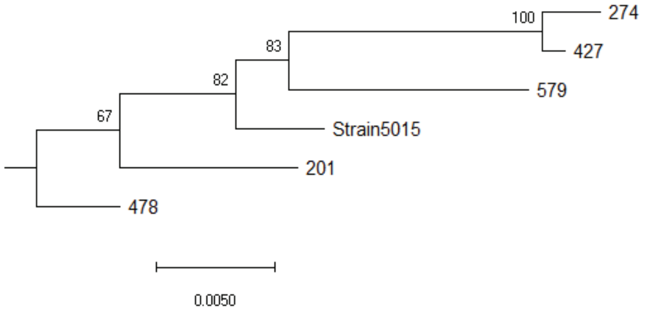

Supplementary Fig. 1. A phylogenetic tree from the concatenated sequence alignment of strain 5015 with all reference STs. The scale bar represent nucleotide substitutions per site.

Supplementary Table 1. MLST profiles of strain 5015 and ST149

Locus	Allele Length (bp)	Allele Strain 5015	Alleles ST149
atpA	378	3	3
dnaE	354	2	2
dnaK	345	**New** (99.42% identity to allele 70)	13
fusA	360	4	35
leuA	384	3	3
odhA	381	2	2
rpoB	342	4	4

Supplementary Table 2. Genes that are unique to strain 5015 and are absent in other 117 *C. diphtheriae* strains

Gene	Function
BUW94_04240	hypothetical protein
BUW94_04375	transposase
BUW94_05795	hypothetical protein
BUW94_05800	hypothetical protein
BUW94_07550	hypothetical protein
BUW94_07555	hypothetical protein
BUW94_07560	hypothetical protein
BUW94_07565	hypothetical protein
BUW94_07570	hypothetical protein
BUW94_07575	hypothetical protein
BUW94_07620	hypothetical protein
BUW94_07625	hypothetical protein
BUW94_07630	hypothetical protein
BUW94_07635	hypothetical protein
BUW94_10700	hypothetical protein
BUW94_11135	hypothetical protein
BUW94_11550	HNH endonuclease
BUW94_11780	50S ribosomal protein L5
BUW94_11845	transposase
BUW94_11850	hypothetical protein
BUW94_11855	hypothetical protein
BUW94_11860	hypothetical protein
BUW94_11865	hypothetical protein
BUW94_11875	hypothetical protein
BUW94_11890	hypothetical protein
BUW94_11915	hypothetical protein
BUW94_12060	hypothetical protein
BUW94_12065	tetracycline resistance MFS efflux pump
BUW94_12070	TetR family transcriptional regulator
BUW94_12075	hypothetical protein
BUW94_12260	transposase

Supplementary Table 3. A list of genes present in strain 5015 but absent in C7 (β) tox +

Gene	Function
BUW94_00005	hypothetical protein
BUW94_00010	hypothetical protein
BUW94_00015	hypothetical protein
BUW94_00020	integrase
BUW94_00025	hypothetical protein
BUW94_00030	hypothetical protein
BUW94_00035	hypothetical protein
BUW94_00040	hypothetical protein
BUW94_00045	hypothetical protein
BUW94_00050	restriction endonuclease
BUW94_00055	restriction endonuclease subunit S
BUW94_00060	hypothetical protein
BUW94_00065	restriction endonuclease subunit R
BUW94_00070	hypothetical protein
BUW94_00075	hypothetical protein
BUW94_00490	hypothetical protein
BUW94_00500	TIGR02611 family protein
BUW94_00620	hypothetical protein
BUW94_01005	hypothetical protein
BUW94_01015	hypothetical protein
BUW94_01340	hypothetical protein
BUW94_01365	hypothetical protein
BUW94_01380	hypothetical protein
BUW94_01565	hypothetical protein
BUW94_01610	hypothetical protein
BUW94_01720	hypothetical protein
BUW94_01725	hypothetical protein
BUW94_01730	hypothetical protein
BUW94_01735	restriction endonuclease
BUW94_01740	hypothetical protein
BUW94_01745	hypothetical protein
BUW94_01750	transposase
BUW94_01755	transposase
BUW94_01840	multidrug DMT transporter permease
BUW94_02025	hypothetical protein
BUW94_02260	hypothetical protein
BUW94_02270	hypothetical protein
BUW94_02275	TIGR02391 family protein
BUW94_02280	restriction endonuclease
BUW94_02285	site-specific DNA-methyltransferase
BUW94_02290	hypothetical protein
BUW94_02295	hypothetical protein
BUW94_02775	transposase
BUW94_02980	DNA repair protein
BUW94_03540	transposase
BUW94_03545	hypothetical protein
BUW94_03585	hypothetical protein
BUW94_03595	transposase
BUW94_03730	DNA-binding protein
BUW94_03820	restriction endonuclease subunit S
BUW94_03840	hypothetical protein
BUW94_04040	iron-sulfur protein
BUW94_04240	hypothetical protein
BUW94_04265	hypothetical protein
BUW94_04305	transposase

Gene	Function
BUW94_04315	hypothetical protein
BUW94_04375	transposase
BUW94_04630	hypothetical protein
BUW94_04635	hypothetical protein
BUW94_04775	hypothetical protein
BUW94_05050	hypothetical protein
BUW94_05420	hypothetical protein
BUW94_05590	hypothetical protein
BUW94_05595	hypothetical protein
BUW94_05600	hypothetical protein
BUW94_05785	transposase
BUW94_05790	IS110 family transposase
BUW94_05795	hypothetical protein
BUW94_05800	hypothetical protein
BUW94_05805	hypothetical protein
BUW94_05810	cell surface protein
BUW94_05855	hypothetical protein
BUW94_05970	hypothetical protein
BUW94_06065	hypothetical protein
BUW94_06070	hypothetical protein
BUW94_06075	hypothetical protein
BUW94_06165	AAA family ATPase
BUW94_06285	hypothetical protein
BUW94_06330	hypothetical protein
BUW94_06340	hypothetical protein
BUW94_06345	hypothetical protein
BUW94_06350	hypothetical protein
BUW94_06360	transcriptional regulator
BUW94_06365	hypothetical protein
BUW94_06370	low molecular weight phosphatase family protein
BUW94_06375	arsenical-resistance protein
BUW94_06380	transcriptional regulator
BUW94_06385	pyridine nucleotide-disulfide oxidoreductase
BUW94_06390	BetI family transcriptional regulator
BUW94_06395	QacE family quaternary ammonium compound efflux SMR transporter
BUW94_06400	QacE family quaternary ammonium compound efflux SMR transporter
BUW94_06405	NIPSNAP family protein
BUW94_06410	alkyl hydroperoxide reductase
BUW94_06415	heavy metal-responsive transcriptional regulator
BUW94_06420	hypothetical protein
BUW94_06425	cytochrome C biogenesis protein
BUW94_06430	redoxin
BUW94_06435	mercury(II) reductase
BUW94_06445	ASCH domain-containing protein
BUW94_06450	hypothetical protein
BUW94_06475	DNA polymerase III subunit epsilon
BUW94_06485	hypothetical protein
BUW94_06490	transposase
BUW94_06500	hypothetical protein
BUW94_06595	hypothetical protein
BUW94_06840	hypothetical protein
BUW94_06845	hypothetical protein
BUW94_06885	hypothetical protein
BUW94_06980	transposase
BUW94_07125	hydrogenase expression protein HupK

Supplementary Table 3… continues.

Gene	Function
BUW94_07325	mycothiol system anti-sigma-R factor
BUW94_07330	hypothetical protein
BUW94_07440	hypothetical protein
BUW94_07525	integrase
BUW94_07530	hypothetical protein
BUW94_07535	hypothetical protein
BUW94_07540	hypothetical protein
BUW94_07545	hypothetical protein
BUW94_07550	hypothetical protein
BUW94_07555	hypothetical protein
BUW94_07560	hypothetical protein
BUW94_07565	hypothetical protein
BUW94_07570	hypothetical protein
BUW94_07575	hypothetical protein
BUW94_07580	hypothetical protein
BUW94_07585	hypothetical protein
BUW94_07590	hypothetical protein
BUW94_07595	hypothetical protein
BUW94_07600	hypothetical protein
BUW94_07605	hypothetical protein
BUW94_07610	hypothetical protein
BUW94_07615	hypothetical protein
BUW94_07620	hypothetical protein
BUW94_07625	hypothetical protein
BUW94_07630	hypothetical protein
BUW94_07635	hypothetical protein
BUW94_07855	oxidoreductase
BUW94_07865	hypothetical protein
BUW94_07965	hypothetical protein
BUW94_08260	hypothetical protein
BUW94_08470	stress-responsive transcriptional regulator
BUW94_08475	hypothetical protein
BUW94_08530	hypothetical protein
BUW94_08630	restriction endonuclease subunit S
BUW94_08640	hypothetical protein
BUW94_08655	transposase
BUW94_08670	hypothetical protein
BUW94_08675	hypothetical protein
BUW94_09025	integrase
BUW94_09030	hypothetical protein
BUW94_09035	hypothetical protein
BUW94_09040	phage tail protein
BUW94_09045	hypothetical protein
BUW94_09050	hypothetical protein
BUW94_09055	hypothetical protein
BUW94_09060	hypothetical protein
BUW94_09065	hypothetical protein
BUW94_09070	hypothetical protein
BUW94_09075	hypothetical protein
BUW94_09080	glycoside hydrolase family 25
BUW94_09085	hypothetical protein
BUW94_09090	hypothetical protein
BUW94_09095	terminase
BUW94_09100	hypothetical protein
BUW94_09105	HNH endonuclease
BUW94_09110	hypothetical protein
BUW94_09115	transcriptional regulator

Gene	Function
BUW94_09260	transposase
BUW94_09265	HNH endonuclease
BUW94_09590	hypothetical protein
BUW94_09655	hypothetical protein
BUW94_09660	hypothetical protein
BUW94_09945	hypothetical protein
BUW94_09980	hypothetical protein
BUW94_10035	hypothetical protein
BUW94_10080	hypothetical protein
BUW94_10105	hypothetical protein
BUW94_10210	site-specific DNA-methyltransferase
BUW94_10290	hypothetical protein
BUW94_10595	hypothetical protein
BUW94_10615	recombinase XerC
BUW94_10620	hypothetical protein
BUW94_10625	hypothetical protein
BUW94_10630	integrase
BUW94_10635	hypothetical protein
BUW94_10640	hypothetical protein
BUW94_10645	hypothetical protein
BUW94_10650	hypothetical protein
BUW94_10670	MFS transporter
BUW94_10675	hypothetical protein
BUW94_10680	acyl-CoA dehydrogenase
BUW94_10685	hypothetical protein
BUW94_10690	3-oxoacyl-ACP synthase
BUW94_10700	hypothetical protein
BUW94_10780	hypothetical protein
BUW94_10805	hypothetical protein
BUW94_10810	hypothetical protein
BUW94_10815	hypothetical protein
BUW94_10820	hypothetical protein
BUW94_10840	hypothetical protein
BUW94_10860	transposase
BUW94_10905	hypothetical protein
BUW94_11025	IS110 family transposase
BUW94_11135	hypothetical protein
BUW94_11140	hypothetical protein
BUW94_11275	hypothetical protein
BUW94_11285	hypothetical protein
BUW94_11380	DUF4244 domain-containing protein
BUW94_11435	hypothetical protein
BUW94_11520	tRNA adenosine deaminase
BUW94_11545	hypothetical protein
BUW94_11550	HNH endonuclease
BUW94_11665	fluoride ion transporter CrcB
BUW94_11685	hypothetical protein
BUW94_11695	DNA-binding response regulator
BUW94_11700	two-component sensor histidine kinase
BUW94_11705	metal transporter
BUW94_11710	copper-translocating P-type ATPase
BUW94_11715	hypothetical protein
BUW94_11780	50S ribosomal protein L5
BUW94_11845	transposase
BUW94_11850	hypothetical protein
BUW94_11855	hypothetical protein
BUW94_11860	hypothetical protein

Supplementary Table 3… continues.

Gene	Function
BUW94_11865	hypothetical protein
BUW94_11870	hypothetical protein
BUW94_11875	hypothetical protein
BUW94_11880	hypothetical protein
BUW94_11885	hypothetical protein
BUW94_11890	hypothetical protein
BUW94_11895	hypothetical protein
BUW94_11900	terminase
BUW94_11905	hypothetical protein
BUW94_11910	hypothetical protein
BUW94_11915	hypothetical protein
BUW94_11920	hypothetical protein
BUW94_11925	hypothetical protein
BUW94_11930	hypothetical protein
BUW94_11935	hypothetical protein
BUW94_11995	transposase
BUW94_12005	hypothetical protein
BUW94_12020	hypothetical protein
BUW94_12030	hypothetical protein
BUW94_12035	hypothetical protein
BUW94_12040	hypothetical protein
BUW94_12050	transposase
BUW94_12060	hypothetical protein
BUW94_12065	tetracycline resistance MFS efflux pump
BUW94_12070	TetR family transcriptional regulator
BUW94_12075	hypothetical protein
BUW94_12095	dihydropteroate synthase
BUW94_12100	hypothetical protein
BUW94_12105	GNAT family N-acetyltransferase
BUW94_12110	NTP-binding protein
BUW94_12175	transposase
BUW94_12200	hypothetical protein
BUW94_12220	IS110 family transposase
BUW94_12260	transposase
BUW94_12285	IS110 family transposase
BUW94_12290	transposase
BUW94_12295	transposase

Supplementary Table 4. A list of genes present in strain C7 (β) tox + but absent in 5015

Gene	Function
CDC7B_0017	hypothetical protein
CDC7B_0024	transposase-like protein
CDC7B_0025	hypothetical protein
CDC7B_0034	transposase-like protein
CDC7B_0038	transposase-like protein
CDC7B_0039	transposase-like protein
CDC7B_0057	hypothetical protein
CDC7B_0065	transcriptional activator
CDC7B_0081	hypothetical protein
CDC7B_0082	hypothetical protein
CDC7B_0083	hypothetical protein
CDC7B_0098	hypothetical protein
CDC7B_0099	putative secreted protein
CDC7B_0101	transposase-like protein
CDC7B_0102	transposase-like protein
CDC7B_0103	hypothetical protein
CDC7B_0104	hypothetical protein
CDC7B_0105	hypothetical protein
CDC7B_0139	hypothetical protein
CDC7B_0140	putative phage integrase
CDC7B_0141	putative transcriptional regulator
CDC7B_0142	hypothetical protein
CDC7B_0143	hypothetical protein
CDC7B_0144	hypothetical protein
CDC7B_0145	putative transcriptional regulator
CDC7B_0146	putative secreted protein
CDC7B_0147	hypothetical protein
CDC7B_0148	hypothetical protein
CDC7B_0149	putative anti-repressor protein
CDC7B_0150	hypothetical protein
CDC7B_0151	hypothetical protein
CDC7B_0152	hypothetical protein
CDC7B_0153	hypothetical protein
CDC7B_0154	hypothetical protein
CDC7B_0155	hypothetical protein
CDC7B_0156	hypothetical protein
CDC7B_0157	hypothetical protein
CDC7B_0158	hypothetical protein
CDC7B_0159	hypothetical protein
CDC7B_0160	putative phage prohead protease
CDC7B_0161	putative phage capsid protein
CDC7B_0162	hypothetical protein
CDC7B_0163	hypothetical protein
CDC7B_0164	hypothetical protein
CDC7B_0165	hypothetical protein
CDC7B_0166	hypothetical protein
CDC7B_0167	hypothetical protein
CDC7B_0168	hypothetical protein
CDC7B_0169	immunity-specific protein Beta241
CDC7B_0170	immunity-specific protein Beta201
CDC7B_0171	immunity-specific protein Beta286
CDC7B_0172	immunity-specific protein Beta371
CDC7B_0173	laminin subunit beta-2
CDC7B_0174	N-acetylmuramoyl-L-alanine amidase
CDC7B_0175	hypothetical protein

Gene	Function
CDC7B_0176	hypothetical protein
CDC7B_0177	putative secreted protein
CDC7B_0178	diphtheria toxin precursor
CDC7B_0183	putative surface-anchored fimbrial subunit
CDC7B_0185	putative surface-anchored fimbrial subunit
CDC7B_0188	putative surface-anchored fimbrial subunit
CDC7B_0190	hypothetical protein
CDC7B_0195	putative excisionase
CDC7B_0196	hypothetical protein
CDC7B_0202	L-idonate 5-dehydrogenase
CDC7B_0203	gluconate 5-dehydrogenase
CDC7B_0204	hypothetical protein
CDC7B_0217	hypothetical protein
CDC7B_0226	transposase-like protein
CDC7B_0282	hypothetical protein
CDC7B_0287	hypothetical protein
CDC7B_0292	hypothetical protein
CDC7B_0293	putative transposase for insertion element
CDC7B_0294	transposase-like protein
CDC7B_0295	hypothetical protein
CDC7B_0299	putative secreted protein
CDC7B_0300	transposase-like protein
CDC7B_0301	transposase-like protein
CDC7B_0302	transposase-like protein
CDC7B_0303	transposase-like protein
CDC7B_0319	hypothetical protein
CDC7B_0392	hypothetical protein
CDC7B_0393	radical SAM domain-containing protein
CDC7B_0394	hypothetical protein
CDC7B_0395	transposase-like protein
CDC7B_0396	hypothetical protein
CDC7B_0397	hypothetical protein
CDC7B_0443	transposase-like protein
CDC7B_0444	hypothetical protein
CDC7B_0452	transposase-like protein
CDC7B_0453	hypothetical protein
CDC7B_0456	hypothetical protein
CDC7B_0457	hypothetical protein
CDC7B_0458	transposase-like protein
CDC7B_0459	hypothetical protein
CDC7B_0464	putative adenine-specific methylase
CDC7B_0478	hypothetical protein
CDC7B_0515	hypothetical protein
CDC7B_0552	putative secreted protein
CDC7B_0575	cell-surface hemin receptor
CDC7B_0581	cell-surface hemin receptor
CDC7B_0586	putative secreted protein
CDC7B_0591	hypothetical protein
CDC7B_0610	transposase-like protein
CDC7B_0613	putative secreted protein
CDC7B_0628	Ni/Fe-hydrogenase B-type cytochrome subunit
CDC7B_0664	hypothetical protein
CDC7B_0684	hypothetical protein
CDC7B_0686	hypothetical protein
CDC7B_0704	hypothetical protein

Supplementary Table 4… continues.

Gene	Function
CDC7B_0705	putative secreted protein
CDC7B_0706	transposase-like protein
CDC7B_0707	transposase-like protein
CDC7B_0709	hypothetical protein
CDC7B_0710	hypothetical protein
CDC7B_0711	hypothetical protein
CDC7B_0712	hypothetical protein
CDC7B_0717	hypothetical protein
CDC7B_0732	enoyl-CoA hydratase
CDC7B_0738	hypothetical protein
CDC7B_0740	hypothetical protein
CDC7B_0741	hypothetical protein
CDC7B_0761	hypothetical protein
CDC7B_0762	hypothetical protein
CDC7B_0764	hypothetical protein
CDC7B_0811	oxidoreductase
CDC7B_0835	transposase-like protein
CDC7B_0836	transposase-like protein
CDC7B_0918	transposase-like protein
CDC7B_0943	hypothetical protein
CDC7B_0982	hypothetical protein
CDC7B_0990	hypothetical protein
CDC7B_1003	hypothetical protein
CDC7B_1026	hypothetical protein
CDC7B_1027	hypothetical protein
CDC7B_1028	hypothetical protein
CDC7B_1029	hypothetical protein
CDC7B_1033	putative integral membrane protein
CDC7B_1042	putative secreted protein
CDC7B_1062	putative secreted protein
CDC7B_1067	tyrosine recombinase XerC
CDC7B_1069	hypothetical protein
CDC7B_1070	hypothetical protein
CDC7B_1071	hypothetical protein
CDC7B_1072	hypothetical protein
CDC7B_1073	hypothetical protein
CDC7B_1074	hypothetical protein
CDC7B_1075	translation initiation factor IF-2
CDC7B_1076	hypothetical protein
CDC7B_1077	putative phage minor capsid protein
CDC7B_1078	hypothetical protein
CDC7B_1079	hypothetical protein
CDC7B_1080	hypothetical protein
CDC7B_1081	hypothetical protein
CDC7B_1082	hypothetical protein
CDC7B_1083	hypothetical protein
CDC7B_1084	hypothetical protein
CDC7B_1085	hypothetical protein
CDC7B_1086	hypothetical protein
CDC7B_1087	minor tail protein Gp26
CDC7B_1088	minor tail protein Gp27
CDC7B_1089	minor tail protein Gp28
CDC7B_1090	gene 29 protein
CDC7B_1091	hypothetical protein
CDC7B_1092	extracellular matrix-binding protein ebhB
CDC7B_1093	hypothetical protein
CDC7B_1094	hypothetical protein

Gene	Function
CDC7B_1095	putative secreted protein
CDC7B_1096	hypothetical protein
CDC7B_1097	hypothetical protein
CDC7B_1098	hypothetical protein
CDC7B_1099	hypothetical protein
CDC7B_1100	hypothetical protein
CDC7B_1101	hypothetical protein
CDC7B_1102	hypothetical protein
CDC7B_1103	hypothetical protein
CDC7B_1104	hypothetical protein
CDC7B_1106	hypothetical protein
CDC7B_1107	gene 64 protein
CDC7B_1108	FAD-dependent thymidylate synthase
CDC7B_1109	hypothetical protein
CDC7B_1110	putative secreted protein
CDC7B_1112	hypothetical protein
CDC7B_1113	hypothetical protein
CDC7B_1114	hypothetical protein
CDC7B_1115	hypothetical protein
CDC7B_1116	hypothetical protein
CDC7B_1117	hypothetical protein
CDC7B_1118	hypothetical protein
CDC7B_1119	hypothetical protein
CDC7B_1120	hypothetical protein
CDC7B_1121	hypothetical protein
CDC7B_1122	hypothetical protein
CDC7B_1123	hypothetical protein
CDC7B_1124	hypothetical protein
CDC7B_1125	hypothetical protein
CDC7B_1126	hypothetical protein
CDC7B_1127	hypothetical protein
CDC7B_1128	hypothetical protein
CDC7B_1129	deoxyuridine 5-triphosphate nucleotidohydrolase
CDC7B_1130	hypothetical protein
CDC7B_1131	hypothetical protein
CDC7B_1132	hypothetical protein
CDC7B_1133	hypothetical protein
CDC7B_1134	putative DNA-binding protein
CDC7B_1135	single-stranded DNA-binding protein
CDC7B_1136	hypothetical protein
CDC7B_1137	hypothetical protein
CDC7B_1138	hypothetical protein
CDC7B_1139	hypothetical protein
CDC7B_1140	hypothetical protein
CDC7B_1141	DNA-damage-inducible protein D
CDC7B_1142	hypothetical protein
CDC7B_1187	putative peptide transport protein
CDC7B_1198	hypothetical protein
CDC7B_1215	putative oxidoreductase
CDC7B_1216	hypothetical protein
CDC7B_1217	putative oxidoreductase
CDC7B_1261	transposase-like protein
CDC7B_1264	hypothetical protein
CDC7B_1275	hypothetical protein
CDC7B_1347	hypothetical protein
CDC7B_1351	L-serine dehydratase
CDC7B_1368	hypothetical protein

Supplementary Table 4… continues.

Gene	Function
CDC7B_1371	transposase-like protein
CDC7B_1375	putative oxidoreductase
CDC7B_1379	putative resolvase
CDC7B_1380	hypothetical protein
CDC7B_1382	hypothetical protein
CDC7B_1383	hypothetical protein
CDC7B_1415	putative transferase
CDC7B_1521	hypothetical protein
CDC7B_1528	ammonium transporter
CDC7B_1538	putative formate/nitrite transporter protein
CDC7B_1540	50S ribosomal protein L5
CDC7B_1573	hypothetical protein
CDC7B_1648	hypothetical protein
CDC7B_1659	hypothetical protein
CDC7B_1660	putative ribonuclease R
CDC7B_1692	transposase-like protein
CDC7B_1711	hypothetical protein
CDC7B_1730	hypothetical protein
CDC7B_1734	hypothetical protein
CDC7B_1745	hypothetical protein
CDC7B_1746	hypothetical protein
CDC7B_1781	hypothetical protein
CDC7B_1797	hypothetical protein
CDC7B_1798	hypothetical protein
CDC7B_1799	transposase-like protein
CDC7B_1839	putative DNA-binding protein
CDC7B_1855	ferrichrome transport system permease protein fhuB
CDC7B_1857	transposase-like protein
CDC7B_1861	hypothetical protein
CDC7B_1862	hypothetical protein
CDC7B_1863	cadmium resistance transporter
CDC7B_1864	putative HTH-type transcriptional regulator
CDC7B_1865	hypothetical protein
CDC7B_1867	putative secreted protein
CDC7B_1868	hypothetical protein
CDC7B_1869	hypothetical protein
CDC7B_1870	transposase-like protein
CDC7B_1871	transposase-like protein
CDC7B_1894	hypothetical protein
CDC7B_1895	PTS system ascorbate-specific transporter subunit IIC
CDC7B_1939	transposase-like protein
CDC7B_1940	hypothetical protein
CDC7B_1951	transposase-like protein
CDC7B_1973	putative surface-exposed virulence protein bigA
CDC7B_1974	putative exported lipase
CDC7B_1975	putative secretory lipase
CDC7B_1978	hypothetical protein
CDC7B_1980	transposase-like protein
CDC7B_1981	hypothetical protein
CDC7B_1983	hypothetical protein
CDC7B_1984	transposase-like protein
CDC7B_1986	transposase-like protein
CDC7B_1988	hypothetical protein
CDC7B_1989	hypothetical protein
CDC7B_1991	hypothetical protein
CDC7B_2007	hypothetical protein
CDC7B_2012	putative secreted protein

Gene	Function
CDC7B_2021	hypothetical protein
CDC7B_2022	transposase-like protein
CDC7B_2023	transposase-like protein
CDC7B_2024	hypothetical protein
CDC7B_2025	hypothetical protein
CDC7B_2026	spermidine/putrescine import ATP-binding protein potA
CDC7B_2027	transposase-like protein
CDC7B_2028	hypothetical protein
CDC7B_2032	hypothetical protein
CDC7B_2033	transposase-like protein
CDC7B_2034	transposase-like protein
CDC7B_2037	hypothetical protein
CDC7B_2038	transposase-like protein
CDC7B_2039	transposase-like protein
CDC7B_2061	transposase-like protein
CDC7B_2064	transposase-like protein
CDC7B_2074	hypothetical protein
CDC7B_2089	hypothetical protein
CDC7B_2101	hypothetical protein
CDC7B_2102	serine/threonine-protein kinase MRCK beta
CDC7B_2104	hypothetical protein
CDC7B_2105	hypothetical protein
CDC7B_2106	hypothetical protein
CDC7B_2107	hypothetical protein
CDC7B_2109	acetyl-CoA thiolase
CDC7B_2110	hypothetical protein
CDC7B_2111	hypothetical protein
CDC7B_2112	hypothetical protein
CDC7B_2113	vibriobactin utilization protein viuB
CDC7B_2114	Fe3+-siderophore ABC transporter ATP-binding protein
CDC7B_2115	Fe3+-siderophore ABC transporter permease
CDC7B_2116	Fe3+-siderophore ABC transporter permease
CDC7B_2117	Fe3+-hydroxamate ABC transporter periplasmic protein
CDC7B_2130	hypothetical protein
CDC7B_2131	hypothetical protein
CDC7B_2136	hypothetical protein
CDC7B_2163	hypothetical protein
CDC7B_2164	hypothetical protein
CDC7B_2165	putative collagen-binding protein
CDC7B_2166	hypothetical protein
CDC7B_2167	hypothetical protein
CDC7B_2168	endo-beta-N-acetylglucosaminidase F2
CDC7B_2189	transposase-like protein
CDC7B_2190	hypothetical protein
CDC7B_2191	hypothetical protein
CDC7B_2192	hypothetical protein
CDC7B_2193	hypothetical protein
CDC7B_2197	hypothetical protein
CDC7B_2198	putative carboxymuconolactone decarboxylase
CDC7B_2199	hypothetical protein
CDC7B_2200	hypothetical protein
CDC7B_2201	LysR-family transcriptional regulator
CDC7B_2203	hypothetical protein
CDC7B_2204	hypothetical protein
CDC7B_2206	type I restriction enzyme S subunit
CDC7B_2207	type I restriction enzyme M protein
CDC7B_2208	type I restriction enzyme EcoKI subunit R

Supplementary Table 4… continues.

Gene	Function
CDC7B_2245	hypothetical protein
CDC7B_2273	putative DNA-binding protein
CDC7B_2287	hypothetical protein
CDC7B_2289	type I restriction enzyme, S subunit
CDC7B_2296	hypothetical protein
CDC7B_2305	putative phosphotransferase system protein
CDC7B_2311	transposase-like protein
CDC7B_2320	hypothetical protein
CDC7B_2324	hypothetical protein

Note: *spaF* gene (CDC7B_0188) has appeared in the gene-set specific to C7 (β) tox + due to limited similarity (32% protein identity) with the *spaF* gene (BUW94_05810) in strain 5015.
